# Surgical Management of Local Recurrences of Renal Cell Carcinoma

**DOI:** 10.1155/2016/2394942

**Published:** 2016-01-26

**Authors:** Ömer Acar, Öner Şanlı

**Affiliations:** ^1^School of Medicine, Department of Urology, Koc University, Istanbul, Turkey; ^2^Istanbul Faculty of Medicine, Department of Urology, Istanbul University, Istanbul, Turkey

## Abstract

Surgical resection either in the form of radical nephrectomy or in the form of partial nephrectomy represents the mainstay options in the treatment of kidney cancer. In most instances, resecting the tumor bearing kidney or the tumor itself provides durable cancer specific survival rates. However, recurrences may rarely develop in the renal fossa or remnant kidney. Despite its rarity, locally recurrent RCC is a challenging condition in terms of the possible management options and relatively poor prognosis. If technically feasible, wide surgical excision and ensuring negative surgical margins are the most effective treatment options. Repeat surgeries (completion nephrectomy, excision of locally recurrent tumor, or repeat partial nephrectomy) may often be complicated, and perioperative morbidity is a major concern. Open approach has been extensively applied in this context and 5-year cancer specific survival rates have been reported to be around 50%. The roles of minimally invasive surgical options (laparoscopic and robotic approach) and nonsurgical alternatives (cryoablation, radiofrequency ablation) have yet to be described. In selected patients, surgical resection may have to be complemented with (neo)adjuvant radiotherapy or medical treatment.

## 1. Introduction

Renal cell carcinoma (RCC) is the most common primary malignancy of the kidney. This tumor accounts for 2% of all cancer diagnoses in humans [1]. Moreover, due to the increasing use of cross-sectional imaging studies, a growing detection rate of incidental renal neoplasms has been described [1]. Based on the current available oncological and quality of life outcomes, localized renal cancers are better managed by partial nephrectomy (PN) rather than radical nephrectomy (RN), irrespective of the surgical approach [2]. Radical nephrectomy is advocated in some patients with localized RCC when PN is unsuitable mainly due to tumor-related and patient-related factors (locally advanced tumor growth, unfavorable tumor location, significant medical comorbidities, etc.) [2].

Local recurrence after nephrectomy for kidney cancer is fairly uncommon. The prevalence has been reported to range between 1 and 2% in different series [3, 4]. The interval between nephrectomy and diagnosis of local recurrence may be quite variable (3 months to 45 years), which highlights the importance of long-term follow-up [5, 6]. Treatment of locally recurrent RCC represents a significant surgical and therapeutic challenge as patients are at high risk for metastatic disease and overall prognosis could be poor. Data on the natural history, patient outcomes, and prognostic factors associated with local recurrence are limited and to date there is no standard management strategy.

Several approaches, such as aggressive surgical excision, radiotherapy, systemic chemotherapy, and observation, have been suggested for the treatment of local recurrence. Among these nonsurgical alternatives, none yielded results superior to surgical excision with negative margins [7–10]. Open surgery is a well-established technique that has been successfully performed for many years [11–13]. Hand-assisted and standard laparoscopic approaches for the resection of isolated RCC recurrences have been reported in limited fashion, and use of robotic instrumentation has yet to be described.

## 2. Methodology

A comprehensive literature review was conducted by entering the keywords of “recurrence,” “nephrectomy,” and “renal cell carcinoma” to the PubMed database. Thereafter, only the articles that were published in the last 10 years and those with eligible follow-up data were taken into consideration.

## 3. Management of Recurrence after Radical Nephrectomy

Recurrent RCC after RN may be a result of metastatic disease within the ipsilateral adrenal gland which was left in situ at the time of the primary surgery, inadequate excision of regional lymph nodes, or recurrent/residual disease in perirenal fatty tissue, in renal fossa, or within the psoas muscle. Inadvertent perioperative tumoral implantation may be another reason. After nephrectomy, locally recurrent disease is defined as recurrent disease in the renal fossa. However, metastasis in the nonresected ipsilateral adrenal gland or lymph nodes makes interpretation of the true incidence of isolated recurrence in the renal fossa difficult.

Despite being rare, solitary recurrences of RCC often have a poor prognosis. However, the time to recurrence may be important, as a longer time before recurrence may improve the likelihood of success with resection. Currently, a 5-year threshold is most commonly used to distinguish early from late recurrences. Although most relapses occur within the first 5 years after surgery, around 10% of local or distant recurrences develop later. Pathological features such as nuclear grade, local extension of primary tumor, and presence of lymphovascular invasion can be helpful to identify patients who will need long-term follow-up (>5 years) in order to detect late recurrences in a timely manner [14].

Systemic treatments appear to be of limited benefit in local recurrence [3]. The relative radioresistance of RCC has limited the role of radiotherapy in managing this disease to the palliation of symptoms [13]. Therefore, surgical removal of isolated local recurrence remains the only possibility of cure in patients with RCC.

There is increasing evidence that wide excision of locally recurrent disease can improve survival. Itano et al., who have analyzed the data of 1737 cases after nephrectomy, reported the incidence of locoregional recurrence as 1.8%. Despite being rare, such event seemed to be related to a poor prognosis; the 5-year survival rate of those who underwent surgical treatment and those who received medical treatment only was 51% and 13%, respectively [3]. In another series of 14 patients with residual disease after RN was treated conservatively, all patients died within a year [15].

The largest series on the treatment of isolated recurrence was published in 2009 by Margulis et al. [4]. Of the 2,945 patients who underwent RN, the authors identified 54 isolated local recurrences in the renal fossa. These however included those in the ipsilateral adrenal and lymph nodes. Adverse prognostic factors were positive surgical margin after resection, size of the recurrence, sarcomatoid histologic features, abnormal serum alkaline phosphatase, and increased lactate dehydrogenase [4]. Patients with 0, 1 and greater than 1 adverse risk features demonstrated cancer specific survival times of 111, 40, and 8 months, respectively. Paparel et al. examined the role of surgery in local RCC recurrence after RN in a multi-institutional study consisting of 12 centers and a total of 72 patients. They found 1-, 3-, and 5-year CSS rates of 74%, 55%, and 46%, respectively. On univariate analysis, time to recurrence and surgical intervention were shown to be the only independent predictive factors for cancer specific mortality [16]. In another recent multi-institutional study, Russell et al. examined 22 patients with isolated ipsilateral nodal recurrence for RCC after RN. All patients underwent complete surgical excision of localized nodal recurrence. Of these cases, 46% progressed to metastatic disease with a median progression-free survival of 12.7 months [17]. Thomas et al. investigated the outcomes of 102 patients with local recurrence treated with surgery from 1990 to 2014. Contrary to the study of Paparel et al., no patients had distant metastatic disease at the time of recurrence surgery. They have calculated the median time from nephrectomy to the diagnosis of recurrent disease as 19 months. Neoadjuvant and salvage systemic therapy was administered in 46 (45.1%) and 48 patients (47.1%), respectively. Despite all these efforts, metastatic progression was observed in 60 patients (58.8%) after a median follow-up of 32 months following recurrence surgery. On multivariate analysis, pathological nodal stage at the time of RN and maximum diameter of retroperitoneal recurrence were identified as independent risk factors for cancer specific death [18].

Although patient series of open surgical resection have been associated with 5-year cancer specific survival of around 50%, perioperative complications may be troublesome. In the study conducted by Thomas et al., which included 102 patients, a total of 30 patients suffered Clavien grade ≥2 complications. Two patients died of multiorgan failure on postoperative days 43 and 45, respectively [18]. Similarly, the incidence of perioperative complications was reported to be 29% (*n* = 14), including 2 grade II, 5 grade IIIa, 3 grade IIIb, 3 grade IV, and 1 grade V, respectively, in the study of Paparel et al. [16].

Exclusively, retrospective noncomparative data exist about this topic, but the available evidence suggests that aggressive local resection offers durable local tumor control and improves survival. Only in cases where complete surgical removal is not feasible due to advanced tumor growth and pain, palliative treatments including radiation treatment should be considered.

### 3.1. Laparoscopic Excision

Open surgical management has been most extensively documented, but the feasibility of laparoscopic management with or without hand assistance has been described only on a limited basis. Nakada et al. were the first to document the outcome hand-assisted laparoscopic resection of local recurrence as a case report [19]. Bandi et al. reported their experience with hand-assisted laparoscopic surgical resection of local recurrence in 5 patients. There was one open conversion in this series. Only 1 of the 4 patients who underwent complete resection recurred locally again during the mean follow-up duration of 43 months and that patient died because of concomitant metastatic disease. The authors suggested that selected patients with low-volume disease not involving adjacent organs should be offered laparoscopic resection [20]. Yohannan et al. reported no open conversion in their laparoscopic RCC recurrence excision series (*n* = 4). Only 1 significant intraoperative complication (diaphragmatic injury) was reported and after a limited follow-up (mean = 12 mo) no recurrences were detected [21]. El Hajj et al. described their experience of 9 patients treated by a pure laparoscopic approach for local recurrence of a renal tumor. They showed that the laparoscopic approach is a safe and feasible alternative treatment option for selected cases with low morbidity [22]. Recently, Sanli et al. reported their experience with laparoscopic excision of locally recurrent RCC. They have identified 5 patients from their institutional laparoscopic database consisting of more than 500 cases. The primary surgical approaches were open radical nephrectomy (*n* = 4) and open partial nephrectomy (*n* = 1). The mean time to diagnosis of recurrence was 51.2 months and the mean size of local recurrence was 3.46 cm. In all patients, a 3-port transperitoneal laparoscopic approach was utilized in order to excise the recurrent mass located in the renal fossa or psoas muscle. There were no significant perioperative complications apart from a laparoscopically managed pleural injury (*n* = 1) and blood transfusion (*n* = 1). At a mean follow-up of 8.4 months, the cancer specific and disease-free survival rates were 100% and 60%, respectively [23].

Table 1 summarizes the main findings in the contemporary open and laparoscopic RCC recurrence surgery series.

### 3.2. Robotic Excision

Robotic surgery is a feasible option for selected patients with isolated RCC recurrence. Gilbert and Abaza recently reported the results of 3 patients who presented with isolated retroperitoneal recurrences of RCC on surveillance imaging up to 5 years following previous nephrectomy, managed via robotic excision. A transperitoneal approach was used in all cases together with intraoperative laparoscopic ultrasonography for tumor localization. There were no open conversions in this series and recurrent RCCs were resected successfully with negative surgical margins. After uneventful postoperative courses, patients were discharged on the first postoperative day. No recurrences were detected with at least 2 years of follow-up [24]. Because of the rarity of such cases, additional long-term studies with larger cohorts are needed to determine the ideal candidates and likelihood of cure.

## 4. Management of Recurrence after Partial Nephrectomy

After nephron-sparing surgical treatment approaches, the recurrent tumoral lesion may develop in the remaining kidney. Venous tumor thrombi or retroperitoneal lymph node metastases may be seen in addition to the recurrent RCC(s). The incidence of recurrence has been reported to be 2.2% after PN for pT1 tumors [2].

Repeat PN may be an option while dealing with recurrences after nephron-sparing approaches. Planning a repeat PN requires a careful balance between renal preservation and oncologic efficacy. Given the added operative challenges and potential postoperative morbidity, few reports are available in the literature. A recent review of 51 planned repeat PNs in 47 patients with locally recurrent disease by Johnson et al. [25] constitutes the largest cohort reported in the literature to date and is illustrative of the complexity of subsequent surgery on the same renal unit. During repeat PN, a more challenging dissection is often encountered due to the alteration of normal tissue planes and perinephric scarring. Not surprisingly, in this study of 51 repeat PNs, there were a total of 40 perioperative complications, with temporary urinary extravasation being the most prevalent. Although the majority did not result in long-term squeal, one patient suffered an intraoperative myocardial infarction and died postoperatively, and 3 patients had the loss of renal unit. The overall major perioperative complication rate was 19.6%, which is higher than the rates reported in PN series of surgically naïve patients. Although there was a statistically significant increase in postoperative serum creatinine (1.35 versus 1.16 mg/dL; *P* < 0.05) and a significant decrease in creatinine clearance (84.6 mL/min versus 95.3 mL/min; *P* = 0.05) and renogram split function (52.3% versus 54.8%; *P* < 0.05), only 3 patients (5.8%) required renal replacement therapy. However, a third of all cases had a solitary kidney and these figures would certainly have been worse if completion nephrectomy had been performed instead of repeat PN. The survival rate seen in this cohort (>95%) is significantly higher than the survival rates of patients who are on hemodialysis or have received a renal transplant [25].

Bratslavsky et al. examined a small cohort of von Hippel-Lindau patients who underwent repeat PN, which they defined as a third or fourth PN on the same renal unit. Major perioperative complications occurred in almost half of the cases and included bowel injury, vascular injury, liver injury, and acute respiratory distress syndrome requiring reintubation. Despite these challenges, more than three-fourths of the operated kidneys were saved, and postoperative changes in renal function were minimal [26]. Repeat PN is a potential therapy for patients with continually recurrent, local kidney cancer, although its application outside the hereditary kidney cancer population and tertiary referral centers may be limited.

## 5. Additional Treatment Modalities

In the era of targeted therapy for locally advanced and metastatic RCC, treatment paradigms using combinations of medical and surgical therapies in patients diagnosed with localized recurrence after nephrectomy are paramount to maximize the oncologic outcome.

Although systemic therapy does not appear to have a primary role in managing local RCC recurrence, Tanguay et al. suggested that it may have a role in conjunction with surgical resection. In their series of 16 patients, 8 received (neo)adjuvant systemic therapy (interferon-based in 7 patients, interleukin-2 + tumor necrosis factor combination in 1 patient). Approximately, the half relapsed at a follow-up of 3–49 months in those receiving systemic therapy. On the other hand, 2 of those treated with surgery alone recurred after 62 and 136 months, respectively [11]. Meanwhile, Margulis et al. not only have confirmed the paramount role of surgical excision of local recurrence, but also highlighted that even in combination with systemic treatment modalities, the probability of progression is over 70% [4].

Patients with positive surgical margins after surgical extirpation were likely to relapse locally and develop distant metastasis. Therefore, these selected groups of patients may benefit from adjuvant systemic therapy. Furthermore, Frydenberg et al. suggested using (neo)adjuvant radiotherapy. Out of 8 patients who were treated with a combination of radiotherapy and surgery, 4 were disease-free at a mean interval of 34 (15–50) months in their series [27].

Besides survival goal, neoadjuvant targeted therapies could potentially downsize recurrent tumor, thus allowing easier surgical resection and potentially reducing tumor spreading risk [28, 29]. In the literature, downstaging has been observed especially for tumors inferior to 5 cm with a 32% decrease in size. [29]. However, in most cases, reduction in tumor size is anecdotal and a general recommendation cannot be made based on this level of evidence.

Newer methods, for example, cryoablation and radiofrequency ablation, may have a role in managing these patients. In their recent article, Monfardini et al. assessed the safety and efficacy of radiofrequency thermal ablation (RFA) for the treatment of retroperitoneal relapse following surgery for RCC [30]. They have ablated a total of 16 lesions in 8 patients with locally recurrent disease after either open radical nephrectomy or open partial nephrectomy. Recurrent lesions were detected after a mean time period of 57 months and the recurrent tumor size varied from 5 to 34 mm. Six patients were treated with the classical percutaneous approach, while laparotomic RFA was deemed necessary for the remaining 2 patients due to the localization of recurrent lesions (anterior pancreatic surface). After a mean follow-up of 12 months, there were no residual enhancements and putting the Clavien grade 2 complications of laparotomic RFA patients aside, no significant complications have occurred during the periprocedural time frame [30]. Nevertheless, the actual role of these minimally invasive treatment options in the management of recurrent RCC remains to be validated. They may be quite useful especially for difficult-to-treat elderly patients with significant comorbidities and those who have recurrent disease despite multiple open surgeries in whom complications are a major concern following recurrence surgeries. Moreover, they may serve as intraoperative adjuncts and can be applied at the same session once wide excision with negative margins seems impossible on laparoscopic or laparotomic exploration.

## 6. Conclusion

Local recurrence after radical or partial nephrectomy is uncommon, but it is associated with poor prognosis. Delay of the onset of recurrence and the applicability of surgical treatment are the major prognostic factors. Wide surgical resection and ensuring negative surgical margin remain the only valid therapeutic options. Open surgical approach has been extensively studied in this context. However, minimally invasive approaches are being investigated with promising early results. The use of (neo)adjuvant therapies in conjunction with surgical treatment in the management of locally recurrent RCC still needs to be defined.

## Figures and Tables

**Table 1:** Outcomes selected contemporary series of surgical experience in the treatment of localized recurrence of RCC (NR: not reported).

	Margulis et al., 2009 [4]	Yohannan et al., 2010 [21]	El Hajj et al., 2013 [22]	Paparel et al., 2014 [16]	Russell et al., 2014 [17]	Thomas et al., 2015 [18]
Number (*n*)	54	4	9	72	22	102

Fuhrman grade of primary tumor (*n*)	Grade 1, 0 Grade 2, 10 Grade 3, 21 Grade 4, 23	Grade 2, 1 Grade 3, 3	Grade 1, 0 Grade 2, 1 Grade 3, 4 Grade 4, 1 Not reported, 3	Grades 1, 0 Grade 2, 2 Grade 3, 35 Grade 4, 15 Unclassified, 7	NR	Grades 1-2, 28 Grades 3-4, 74

T stage of primary tumor (*n*)	T1, 10 T2, 11 T3, 33 T4, 0	T1, 1 T2, 2 T3, 1	Tx, 3 T1, 3 T2, 1 T3, 2 T4, 0	Tx, 2 T1, 21 T2, 13 T3, 32 T4, 4	T1, 6 T2, 4 T3, 12 T4, 0	T1, 20 T2, 20 T3, 59 T4, 3

Mean/median age at time of recurrence (yrs.)	NR	57	67	NR	62	55

Mean/median time to recurrence (mos.)	10	11.5	83	26.5 ± 3.3	31.5	19

Mean/median size of recurrence (cm)	6	5.7	3.4	4.7 ± 0.5	2.6	4.5

Neoadjuvant therapy (*n*)	27	NR	3	2	3	46

Surgical approach for recurrence surgery (*n*)	Open	Laparoscopic	Laparoscopic	Open, 47 Laparoscopic, 1	Open, 21 Laparoscopic, 1	Open, 99 Laparoscopic 3

Mean/median operative time (min.)	377.5	195	144	133.2	NR	210

Mean/median estimated blood loss (mL)	600	187.5	430	427.5	300	700

Mean/Median length of hospital stay (day)	7	2.5	4.5	NR	5	7

Positive surgical margin (*n*)	NR	0	0	NR	1	12

Complications (*n*)	Clavien 3 and 4, 8	Clavien 1, 1	Clavien 1, 3 Claiven 3b, 1	Clavien 2, 2 Clavien 3a, 5 Clavien 3b, 3 Clavien 4, 3	Clavien 3b, 2	Clavien 1, 16 Clavien 2, 15 Clavien 3, 12 Clavien 4, 1

Perioperative mortality (*n*)	2	0	0	1	0	2

Adjuvant therapy after recurrence surgery (*n*)	16	NR	2	0	Targeted therapy, 2 Radiotherapy, 1	48

Follow-up (months)	NR	38	38	51.7 ± 4.3	22.2	32

Survival data	Cancer specific survival: 11 months	Disease free survival: 75% Cancer specific survival: 100%	Disease free survival: 67% Cancer specific survival: 89%	Cancer specific mortality: 39%	Median progression free survival: 12.7 months	Median progression free survival: 23 months Median cancer specific survival: 66 months
